# Reconsidering calling 911: Is it time to set a new standard for mental health crisis response?

**DOI:** 10.1371/journal.pmen.0000084

**Published:** 2024-08-02

**Authors:** Rupinder K. Legha

**Affiliations:** Founder, Antiracism in Mental Health Fellowship, Los Angeles, California, United States of America; PLOS: Public Library of Science, UNITED KINGDOM OF GREAT BRITAIN AND NORTHERN IRELAND

This opinion piece critiques the standard recommendation of calling 911 or going to your closest emergency room during a mental health emergency. Based on her professional and lived expertise, the author contends that this standard does not weigh the risks of police involvement, the carceral nature of mental health crisis response, and how these experiences breed stigma and shame, leaving lasting wounds, which keep people from seeking mental healthcare in the future. This opinion piece closes by offering here and now strategies for how healthcare providers and people accessing mental health care can promote a new standard of care for mental health crisis management, one that promotes healing and protection, rather than risking additional harm.

In the United States (U.S.), there have been cases in which police have shot people seeking emergency assistance for a mental health crisis. Therefore, calling 911 during a mental health emergency is not necessarily the safest option. Indeed, it can be potentially fatal. On March 9th, 2024, family members of Ryan Gainer [1], a Black teenager with autism, called 911 for the sixth time in weeks. Within moments of arriving, San Bernardino County sheriff’s deputies shot and killed Ryan, who was running towards them carrying a gardening tool. Several weeks later, 19-year-old Wiz Rosario [2], whose family immigrated from Bangladesh a decade ago, called 911 seeking help while in distress. His brother contested police reports, noting that his mother was restraining Wiz–who had pulled out a pair of scissors–when officers opened fire [2].

Remarkably, calling 911 or going to your closest emergency room is a standard recommendation during a mental health crisis, one backed by major mental health organizations [3]. I am on the view that these standards [4] do not weigh the risks of police involvement. This erasure is glaring because the police have become the default responders for mental health emergencies in the U.S.; and when they do come, the risk of fatality is especially high for racially minoritized people. Specifically, almost half of the people who die at the hands of police have some kind of disability [5], and more than half of disabled African-Americans have been arrested by the time they turn 28—double the risk in comparison to their white disabled counterparts [6].

Even when individuals successfully access mental health care in emergency room settings, quality care is not guaranteed. On March 22nd, 2020, University of Rochester Medical Center providers quickly discharged Daniel Prude [7], despite his family’s pleas for help and reports of multiple recent suicide attempts, including jumping in front of a train. After family members called 911 in crisis later the same day, police forcefully subdued Prude and through this, he passed due to the resultant asphyxiation. Yet the University of Rochester Medical Center publicly asserted they provided “medically appropriate and compassionate care” [8]. This instance exemplifies the need for new practices during mental health emergencies, that at a minimum, reduce the risk of death.

This new standard must account for the litany of humiliations emergency room settings expose people to, including sitting for hours or days with little to no care or being forcibly restrained or injected with sedating medications [9]. Nurses and doctors, like police officers or anyone else, are susceptible to racist tropes, leading them to sometimes perceive racially minoritized people as dangerous and threatening when they express distress or need help. Emergency care settings, often chaotic and understaffed, create an environment in which medical providers may feel under threat and with no option but to restrain someone who is threatening others. However, I am of the view that this intervention is not always done with the intention of preventing violence. Rather, it could be viewed as an act of violence, a form of medical brutality and an abuse of power intended to control and subdue people [10]. Much like police violence, this coercive clinical intervention can go from being an option of last resort to an option of convenience. Instead of firing shots, healthcare providers tie people down and inject them [11]. Injuries are not unheard of, and the emotional toll of these traumatic encounters is immeasurable [12].

I have witnessed this injustice repeatedly as a psychiatrist working in these settings. I recognize that the providers working in these settings, which are often overwhelmed by the number of people urgently needing help, feel like they have no choice. However, this helplessness cannot justify emergency room settings worsening mental health crises, rather than improving them. It cannot numb us as healthcare providers to the pain and trauma people experience as a result of our interventions. I believe we have to take responsibility for how, by breeding fear, exclusion, and isolation, mental health crisis response experiences deepen stigma and shame, leaving lasting wounds, which keep people from seeking mental healthcare in the future [13].

I feel strongly about this because I know this heartache firsthand. Over two decades ago, I was psychiatrically hospitalized several times over the course of a year while moving in and out of mental health crises. Though I was never forcibly injected or restrained nor had the police called on me, the threat of it loomed. I cannot imagine how devastated I would have been had it actually happened. Would I have survived? It took me years to move past the trauma, shame, and alienation of the threat alone. The medical and psychiatric professions’ entrenched ableism prevented me from speaking openly about my experience, and I was repeatedly discriminated against during my training. For a long time, this stigma felt like a permanent stain; but it quickly faded once I turned my pain into power. As part of the oath I took as a doctor, I vowed never to perpetrate these harms against the people I cared for. I will not do to others the things I would not want done to myself, especially during life’s most vulnerable moments.

This vow and the deaths of Ryan Gainer, Wiz Rosario, Daniel Prude, and so many others, inspired me to establish a new standard of care for mental health emergencies that minimizes harm and avoids death (see Table 1).

**Table 1:** How healthcare providers and people accessing mental healthcare can promote a new standard of care for mental health crisis management.

**Healthcare Providers:** The following recommendations align with the Antiracism in Mental Health’s Think, Reflect, Act framework for reimagining mental health standards of care with an antiracist lens to challenge mental health racial inequities.
1. 1. Think: • Learn about mental healthcare’s intersection with the carceral state • Learn major racial health inequities pertaining to the behavioral health emergency continuum of care. • Learn more about social movements (e.g. Black Emotional and Mental Health • Collective), including disability justice (e.g. Sins Invalid), and their visions for more just and humane options for mental healthcare. 2. Reflect: • Engage in reflective practices regarding what it would be like for you to experience the behavioral health emergency continuum of care. • Consider accompanying one of the people you care for as they proceed through this journey, so you understand what you are asking of people when you make clinical recommendations 3. Act: • Give “the full informed consent” by communicating the risks and harms involved across the behavioral health emergency continuum of care (police, coercion, stigma, trauma). • Co-create with people receiving care an individualized crisis management plan, weighing the risks and benefits for implementing existing standard recommendations. Consider involving family, neighbors, and friends, • Write letters of support for people who do go to the emergency room outlining key aspects of recent history and recommendations for responsive, humane care, and ask that this documentation be included in the chart. • Consider outreaching the police in advance if someone is known to have mental health emergencies. Consider doing neighborhood outreach, as well, so neighbors know not to immediately resort to calling the police if crises arise. • Look into local peer respite centers and community action teams providing alternatives to calling 911 during mental health emergencies
**Members of the Community:** The following recommendations position people to be protected against the harms baked into standard crisis management for mental health emergencies.
1. Think: • Learn more about Daniel Prude’s story to understand why calling 911 or going to an emergency room during a mental health crisis can be dangerous. 1. Reflect • Reflect on an ideal vision for mental healthcare during an emergency. What would it look like and how would it feel? • Calling 911 can feel like a relief, particularly if family members feel helpless during mental health emergencies. Reflect on the fear families feel in the midst of these crises. 2. Act: • Hold mental health providers accountable for providing humane care that acknowledges and accounts for risks in the behavioral health crisis continuum of care as part of their oath by asking the following questions at the beginning of care *1*. *Can you tell me what to do during a psychiatric emergency for my loved one*? *2*. *Can you explain the risks and benefits involved with these interventions*? *3*. *How do you determine whether the benefit of calling 911 and going to the emergency room outweighs the risk of further violence and trauma*? *4*. *Do you determine whether the benefit outweighs the risks at all*? *5*. *Is it possible to contact a mobile crisis team or a community action team?* • Consider forming community action teams that can help respond to mental health crises without police involvement. • Educate neighbors about loved ones who frequently experience mental health emergencies and explain the risks of calling the police or 911.

Giving families what I call “the full informed consent”—a more detailed account of the often-unstated risks baked into mental health “care”—is a central tenet. The “full informed consent” is a term I coined to describe the process of giving children, adults, and families engaged in the mental healthcare system a clearer depiction of the harms baked into this system. This term is a play on the term “informed consent,” which healthcare providers are required to provide when offering a medical intervention. Full informed consent implies that the full truth about the mental healthcare system’s harms–for example related to police involvement, coercion, and racism–is not formally accounted for by this required process. I tell all families–and racially minoritized families in particular: “If you or your child is having a mental health emergency, the *standard* recommendation is to call 911 or go to the closest emergency room. However, getting the police involved carries significant risks, including getting shot and killed. While we weigh the risks and benefits of this option, let’s look at other ones, too, so you can make an informed decision.” Another core tenet involves keeping people at home, where their families–rather than the police or healthcare providers–can care for and monitor them along with medical support from me. If families do elect to go that more traditional route, I write a doctor’s letter detailing my firm direction to avoid force, provide quality care, and contact me. The letter, which captures a third core tenet related to what I call “clinical activism,” sends a clear message that the family and their provider are watching the care closely. It promotes accountability.

This new standard of crisis management introduces new opportunities—and new challenges for all parties involved. Families might not feel safe supporting someone at home during a crisis, particularly if they are bigger or stronger and have been violent before. Providers may not feel equipped, supported or even willing to engage practices far from the norm. They may be legitimately concerned about protecting their own emotional wellbeing while being asked to provide services that demand too much from them professionally. But while this stay-at-home approach does require significant investment of time, effort, and money, it can actively prevent the loss of life, which is priceless, and it can help people get better faster, knowing they will not be subjected to the indignities of the behavioral health crisis continuum of care. I have witnessed this time and time again. If this approach became the new standard, teams of providers could work together with communities to make it more sustainable. There is joy and freedom on the other side if we, as providers, commit to the mantra, “I will not do to others the things I do not want done to myself.”

Some of this work is already underway. Through the Antiracism in Mental Health Fellowship [14], a growing circle of practitioners is coming together to innovate protective standards incorporating real-world risks, like police violence, rather than ignoring them the way standard approaches do. It specifically reviews how to implement stay-at-home strategies while avoiding calling 911 and going to emergency rooms. Its “think, reflect, act” framework compels providers to acquire knowledge domains often overlooked by the prevailing biomedical model, including histories of oppression and mental healthcare’s intersection with the carceral state (think). Journaling exercises and group discussions guide providers to confront the harm they have caused through “care” (reflect), while antiracist action steps galvanize them to stand up in defense of the people for whom they care (act). My lived experience surviving the mental health system is my most precious expertise that informed the fellowship’s approach, particularly the emphasis on self-reflection. We cannot shield people from harm until we viscerally connect to the pain this harm causes. A companion fellowship geared towards the community is under development. It will offer individuals and families knowledge and support so they can participate in this new standard for managing emergencies.

While these efforts are promising, only when training guidelines and clinical practice standards mandate them will this new standard become the norm. Perhaps, costly settlements will provide motivation. The City of Rochester recently agreed to pay 12 million dollars to settle a wrongful death lawsuit filed by Daniel Prude’s family. Attorneys for Prude’s estate are calling upon the New York Legislature to pass a bill that would replace police response to calls for mental health interventions with trained mental health professionals [15]. Replacing the police with mental health providers, however, does not account for the other harms embedded in the crisis continuum of care.

For now, the more immediate strategies described in Table 1 can prevent another death at the hands of the police when people in the throes of emotional despair need help. As healthcare providers, we can no longer stand by hands in the air while another death moves in and out of the news cycle. We owe people more than this. As community members, there are things we can do to protect ourselves and our loved ones. Is It time to set a new standard for mental health crisis response? Yes, I believe that time is now.

## References

[1] Gorman S. Fatal police shooting of autistic boy in California investigated. Reuters. 2024 Mar 23. Available at https://www.reuters.com/world/us/fatal-police-shooting-autistic-boy-california-investigated-2024-03-13/

[2] Shanahan E. Police fatally shoot Queens man who brandished scissors, officials say. NY Times. 2024 Mar 27. Available at https://www.nytimes.com/2024/03/27/nyregion/win-rozario-queens-nypd.html

[3] American Academy of Child and Adolescent Psychiatrists. What is a psychiatric emergency? Reviewed July 2018. Available at https://www.aacap.org/AACAP/Families_and_Youth/Facts_for_Families/FFF-Guide/What_is_a_Psychiatric_Emergency_126.aspx.

[4] National Institute of Mental health. Help for mental illness. Available at https://www.nimh.nih.gov/health/find-help#:~:text=In%20life%2Dthreatening%20situations%2C%20call,with%20a%20trained%20crisis%20counselor.

[5] The Ruderman Foundation. White paper on media coverage of law enforcement use of force and disability, a media study (2013–2015) and overview. 2016. Available at https://rudermanfoundation.org/wp-content/uploads/2017/08/MediaStudy-PoliceDisability_final-final.pdf

[6] McCauley EJ. The cumulative probability of arrest by age 28 years in the United States by disability status, race/ethnicity, and gender. Am Journal of Pub Health. 2017 Dec 1;107(12):1977–1981. doi: 10.2105/AJPH.2017.304095 29048954 PMC5678390

[7] Dahlberg B. Rochester hospital released Daniel Prude hours before fatal encounter with police. National Public Radio. 2020 Sept 29. Available at: https://www.npr.org/sections/health-shots/2020/09/29/917317141/rochester-hospital-released-daniel-prude-hours-before-fatal-encounter-with-police.

[8] Dahlberg B. New York investigates Daniel Prude’s stay at Strong Memorial Hospital. National Public Radio. 2020 Sept. 29. Available at https://www.npr.org/2020/09/29/918101554/new-york-investigates-daniel-prudes-stay-at-strong-memorial-hospital.

[9] Eswaran V, Molina MF, Hwong AR, Dillon DG, Alvarez L, Allen IE, et al. Racial disparities in emergency Department physical restraint use: A systematic review and meta-analysis. JAMA Intern Med. 2023;183(11):1229–1237. doi: 10.1001/jamainternmed.2023.4832 37747721 PMC10520842

[10] MedLine Plus. Use of restraints. Available at https://medlineplus.gov/ency/patientinstructions/000450.htm#:~:text=Patient%20Rights&text=Restraints%20should%20be%20used%20only,to%20the%20patient%20or%20caregiver.

[11] Evans D, Wood J, Lambert L. Patient injury and physical restraint devices: a systematic review. J Adv Nurs. 2003 Feb;41(3):274–82. doi: 10.1046/j.1365-2648.2003.02501.x . 12581115

[12] Da Silva AG, Baldaçara L, Cavalcante DA, Fasanella NA, Palha AP. The impact of mental illness stigma on psychiatric emergencies. Front Psychiatry. 2020 Jun 19;11:573. doi: 10.3389/fpsyt.2020.00573 ; PMCID: PMC7319091. 32636773 PMC7319091

[13] Legha R. Medical brutality, social media, and collective activism. Kevin MD. Rupinder. October 29, 2020. Available at https://www.kevinmd.com/2020/10/medical-brutality-social-media-and-collective-activism.html

[14] Antiracism in Mental Health Fellowship. www.rupileghamd.com

[15] Lahman S and Canne K. Rochester to pay $12M to settle lawsuit filed by Daniel Prude family, largest civil rights settlement in city’s history. Rochester Democrat and Chronicle. October 6, 2022. Available at: https://www.democratandchronicle.com/story/news/2022/10/06/daniel-prude-death-lawsuit-rochester-ny-12-million-settlement/69543956007/

